# A novel technique to minimize facial pressure ulcers during prone surgery: A randomized controlled trial

**DOI:** 10.1097/MD.0000000000043275

**Published:** 2025-09-12

**Authors:** Mehmet Nuri Erdem, Yigit Kultur

**Affiliations:** a Yeni Yuzyil University Gaziosmanpasa Hospital, Orthopedics and Traumatology, Istanbul, Turkey.

**Keywords:** deformities, degenerative pathologies, facial pressure ulcer, prone position, spinal surgery

## Abstract

**Background:**

The prone position is widely used in spinal surgery. However, facial pressure ulcers (FPU) associated with the prone position are a significant cause of postoperative morbidities. Numerous methods have been described for preventing these lesions; however, practical and universally applicable solutions remain elusive. A novel approach has been described that incorporates cast padding placed over prone pads with extensive facial draping in an attempt to reduce the incidence of FPU. The purpose of this study is to evaluate the effectiveness of this preventive measure in reducing the incidence of FPU in patients undergoing prolonged spinal procedures in the prone position.

**Methods:**

This was a prospective, randomized trial. Patients undergoing spine surgery and staying in a prone position for more than 6 hours were included. All patients were evaluated in the early postoperative period, and clinical follow-up was done for at least 6 months after surgery to monitor for potential complications and delayed wound healing. Patients were randomized into 2 groups: group A received standard anesthesia preparation with routine head positioning, while group B used prone pads wrapped with cast padding and had the patients’ faces covered by a surgical drape. In addition, the results were analyzed based on surgical indications and categorized into degenerative and deformity subgroups. The FPU was classified according to the National Pressure Ulcer Advisory Panel Staging System.

**Results:**

The study included 29 and 28 patients in groups A and B, respectively. The FPU rate was significantly higher in group A (82.8%) than in group B (57.1%) across both degenerative (group A, 75.0%; group B, 55.0%) and deformity subgroups (group A: 100%, group B: 62.5%; *P* = .045). Notably, the FPU rates in the deformity subgroup (82.4%) exceeded those in the degenerative subgroup (65.0%); however, the difference was not statistically significant (*P* = .224).

**Conclusion:**

Wrapping prone pads with cast padding and fully covering the face with a surgical drape is a simple, time-efficient, and universally applicable method that significantly reduces the incidence of FPU associated with the prone position.

## 1. Introduction

The prone position is widely used in spine-related surgical procedures. In the literature, complications associated with the prone position under anesthesia range between 5% and 66%.^[1]^ Proper positioning of the patient on the operating table with gel pads or frames, as well as protection of areas under pressure, is crucial to prevent possible pressure complications. Facial complications were the most frequent and significant complications. Although rare, vision loss, one of the most severe complications, can occur in 0.05% to 1% of cases.^[2]^

Facial pressure ulcers (FPU), one of the most common pressure complications, do not leave scars and heal without causing permanent damage. However, FPU increase morbidity, hospital stay duration, and cost.^[3–5]^ More importantly, FPU negatively affect the morale and recovery motivation of patients undergoing surgery for spinal pathologies. FPU occur more frequently after prolonged surgeries. These lesions, which range from edema to skin necrosis, have led to the development of various preventive methods.

An important risk factor is direct pressure to the facial area as a result of poorly designed or misaligned headrests. Cunha et al^[6]^ point out that horseshoe-shaped headrests must be avoided as they create focal pressure on ocular globes and facial soft tissues and predispose them to injury. They recommend using foam pillows with eye, nose, and mouth apertures because their design allows for even pressure distribution and reduces localized tension to skin.

Techanivate et al^[7]^ present findings indicating the occurrence of FPU in 82 out of 300 patients (27.3%) and surmise that minimizing surgery time, blood loss, and replacement fluids may help in preventing the ulcers. The authors further observe that variation in padding and positioning styles can make a wide difference in incidence rates.

Besides, Grisell and Place^[8]^ compared three types of face pads and concluded that the ROHO dry flotation device (The ROHO Group, Belleville) imposed the least face pressure. Also, Koreckij et al^[9]^ recommended the use of Gardner-Wells tongs to eliminate the pressure on the face with a 45° angle and 15 lb. traction on the head. Kadam et al^[10]^ reported that the use of 2 attachments adapted to the operating table with a cervical tong could eliminate the complications of facial pressure during posterior cervical and thoracic spine surgeries. Other studies have shown that Mayfield clamps or Gardner-Wells tongs can also reduce the risk of developing an FPU.^[11]^

However, these applications are invasive and associated with considerable consumptions of time. In contrast, the method being presented here is much simpler and less time-consuming. It can, more practically, be applied for all procedures in the prone position, not only for those expected to be long or under spinal surgery, without needing any additional equipment.

This study aimed to evaluate and compare the incidence of FPU in patients who underwent spinal surgery in the prone position for more than 6 hours. The hypothesis of this study is that these lesions can be prevented or minimized through simple measures.

## 2. Materials and methods

### 2.1. Study design and participants

This prospective randomized study, approved by the Ethics Committee of Yeni Yüzyil University (approval number. 2022/02–812), included patients over 50 years of age who underwent posterior instrumentation and fusion surgery for spinal pathologies (deformities or degenerative conditions) at the thoracic or lumbar spine levels between April 2022 and September 2023. Participants were randomly assigned to 2 groups using an automated randomization process generated through computer software designed in collaboration with an online randomization website: https://www.randomizer.org/. Randomization was conducted prior to surgery by an impartial researcher who was not involved in treatment of patients or assessment of outcomes.

The study comprised a cohort of 57 participants, all of whom underwent spinal surgery while prone for more than 6 hours. These patients were evaluated in the early postoperative period, and clinical follow-up was done for at least 6 months after surgery to monitor for potential complications and delayed wound healing. The patients were informed of their participation in the study. Informed consent was obtained from the patient for publication of this study. However, they were not informed about the group to which they were assigned. Patients with preoperative facial lesions, wounds, or skin conditions were excluded from this study.

### 2.2. Interventions

Two new prone pads (Prostrate Head Pads; Auckland Medical Polymer (Tianjin) Co. Ltd., Tianjin, China) were used, and all surgeries were performed using these pads. The senior surgeon (MNE) and anesthesiologist jointly positioned the patients’ heads during surgery.

**Figure 1.**
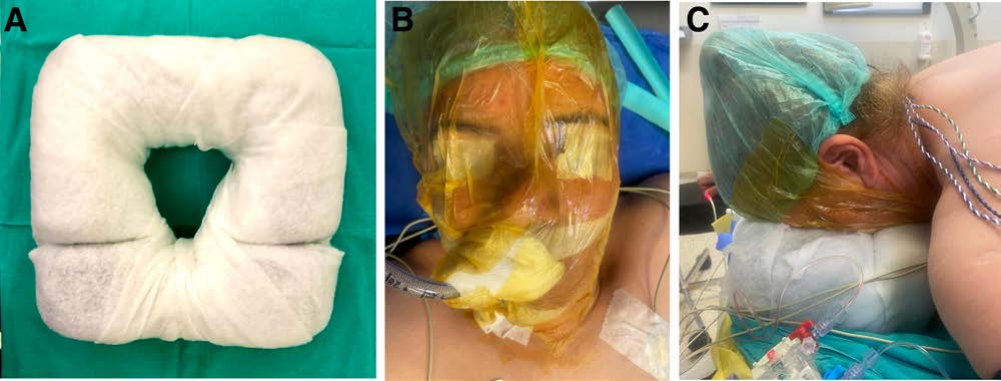
(A) Preparation of the prone pad in the operating room, (B) covering the patient’s face with a surgical drape, and (C) positioning on the prone pad.

The following groups were formed:

- Group A (standard method): The face was positioned by the anesthesiology team using routine methods. After intubation and catheterization, Bepanthen cream (Bayer, Leverkusen, Germany) was applied to the patient’s face and then directly placed on the prone pad.
- Group B (modified method): Two-layer cast padding was wrapped around the prone pad before positioning the face (Fig. 1A). After intubation and catheterization, the patient’s face was fully covered with a drape (3M Co., Saint Paul) and Bepanthen cream was applied over the drape (Fig. 1B). The face was then placed on a padded headpad (Fig. 1C).

### 2.3. Outcome measures

Facial lesions observed 24 hours postoperatively were monitored regularly, with the most severe lesion stage recorded for analysis. FPU were classified based on the National Pressure Ulcer Advisory Panel Staging System.^[12]^ For statistical convenience, patients without lesions were categorized as stage 0.

- Stage 0: No lesion
- Stage 1: Non-blanchable erythema of intact skin
- Stage 2: Partial-thickness skin loss involving the epidermis and/or dermis
- Stage 3: Full-thickness skin loss with damage or necrosis of subcutaneous tissue that may extend down to, but not through, the underlying fascia
- Stage 4: Full-thickness skin loss with extensive destruction, tissue necrosis, or damage to the muscle, bone, or supporting structures.

### 2.4. Statistical analysis

Categorical data are presented as frequencies and percentages, whereas continuous data are summarized as means and ranges. The Kolmogorov–Smirnov test was used to assess sample distributions of age, sex, and prone position duration between groups A and B. Contingency tables were created to compare FPU incidence and staging between the groups, with statistical significance tested using the exact chi-square test.

Cramer’s *V* was calculated to determine the effect sizes, with thresholds of 0.10, 0.30, and 0.50, indicating small, medium, and large effects, respectively. Adjusted Pearson residuals were used for row comparisons, with *P*-values derived from *Z*-scores. Statistical significance was defined as *P* < .05, and all analyses were performed using Statistical Package for the Social Sciences (SPSS) version 25.0 (IBM Corp., Armonk).

Cramer’s *V* was calculated to determine effect sizes. The decision criteria for Cramer’s *V* were as follows: degrees of freedom were considered small for 0.10, medium for 0.30, and large for 0.50. For row comparisons of contingency tables, adjusted Pearson residuals were calculated. As adjusted Pearson residuals followed a standard normal distribution, *P*-values were calculated on *Z*-scores. Statistical tests with *P* values <.05 were considered significant. All statistical analyses were performed using SPSS 25.0.

## 3. Results

The study initially included 60 patients; however, 57 patients completed the study. One patient from each group required reoperation within the first 72 hours postoperatively and was subsequently excluded. Additionally, 1 patient in group B was excluded because of the development of facial herpes zoster. Therefore, the final analysis included 29 patients in group A and 28 patients in group B.

**Figure 2.**
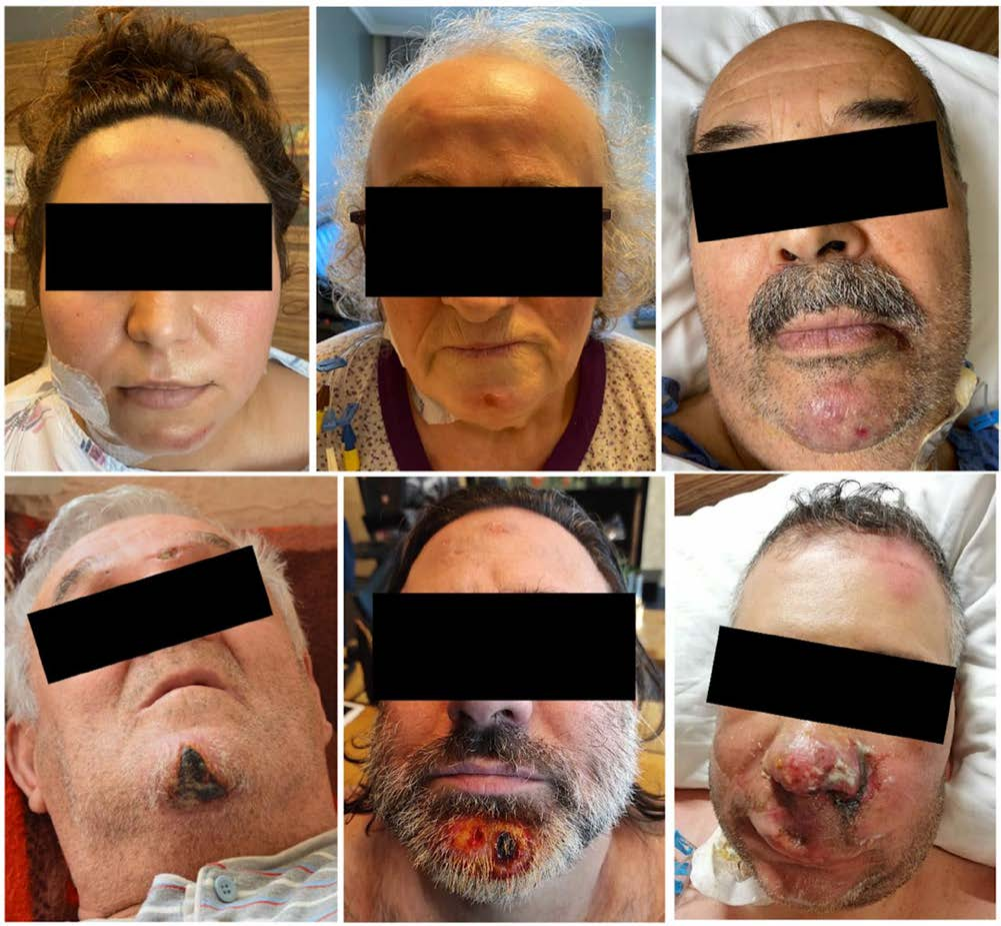
Appearance of facial pressure ulcers of varying stages.

In group A 20 patients underwent surgery for degenerative pathologies and 9 for adult deformities. Similarly, in group B, 20 patients had degenerative pathologies, and 8 had adult deformities. All 57 patients were followed up sufficiently to stage the facial lesions (Fig. 2), and no complications occurred that necessitated termination of the study. The mean age was 62.2 years (range: 51–74 years) and 63.5 years (range: 50–78 years) in groups A and B, respectively. The mean prone position durations were 425 minutes (range: 350–660 minutes) in group A and 438 minutes (range: 360–720 minutes) in group B, with no significant differences in age, sex, or duration between the groups (Table 1).

**Table 1:** Descriptive statistics of the patients.

	Group A (n = 29)	Group B (n = 28)	*P*
Age (yr)	62.17 (51–74)	63.54 (50–78)	.827
Gender			
Female	18 (62.1%)	15 (53.6%)	.596
Male	11 (37.9%)	13 (46.4%)	
Prone position duration (min)	424.83 (350–660)	437.50 (360–720)	.986

Continuous data are presented as mean (min–max). Categorical data shown as n (%). *P* values were calculated using the Kolmogorov–Smirnov (exact) test.

In this study, the FPU rate was 70.2% among all patients. When comparing the 2 groups, the FPU rate was significantly higher in group A (82.8%) than in group B (57.1%; *P* = .045). Furthermore, in both the degenerative (group A: 75.0%, group B: 55.0%) and deformity (group A: 100%, group B: 62.5%) subgroups, the FPU rate was higher in group A; however, the difference was not statistically significant (*P* = .32, *P* = .082). For the degenerative and deformity groups, the FPU rate in the deformity group (82.4%) was higher than that in the degenerative group (65.0%); nonetheless, the difference was not statistically significant (*P* = .224; Table 2).

**Table 2:** Incidence of facial pressure ulcers among patients.

	Group A (n = 29)	Group B (n = 28)	Significance (effect size)
FPU			
No	5 (17.2%)	12 (42.9%)	.045* (.280—small)
Yes	24 (82.8%)	16 (57.1%)	
	Degenerative patients only (n = 20)	Degenerative patients only (n = 20)	
FPU			
No	5 (25.0%)	9 (45.0%)	.32 (.210—small)
Yes	15 (75.0%)	11 (55.0%)	
	Deformity patients only (n = 9)	Deformity patients only (n = 8)	
FPU			
No	0 (0%)	3 (37.5%)	.082 (.491—medium)
Yes	9 (100.0%)	5 (62.5%)	
	Degenerative group (n = 40)	Deformity group (n = 17)	
FPU			
No	14 (35.0%)	3 (17.6%)	.224 (.174—small)
Yes	26 (65.0%)	14 (82.4%)	

Data are shown as n (%). *P* values were calculated using Fisher’s exact test. Cramer’s *V* was calculated to determine effect size.

FPU = facial pressure ulcer.

* *P* < .05.

National Pressure Ulcer Advisory Panel Staging System staging assessments revealed significant differences between the groups (Table 3). Specifically, when evaluating all patients, a higher proportion of patients without FPU (stage 0) was observed in group B (*P* = .035), whereas the proportion of patients in stage 3 was significantly greater in group A (*P* = .042). In the degenerative group, comparisons between groups A and B indicated higher rates of FPU in stages 1, 2, and 3 in group A, although these differences were not statistically significant (*P* = .525, P =. 677, *P* = .311). Similarly, in the deformity group A, group A exhibited higher rates of FPU in stages 2 and 3 than in group B; however, this difference was not statistically significant (*P* = 402, *P* = .072), whereas stage 0 was significantly more prevalent in group B (*P* = .043).

**Table 3:** Distribution of facial pressure ulcers by stage.

	Group A (n = 29)	Group B (n = 28)	Significance	Overall significance (effect size)
Stage				
0	5 (17.2%)	12 (42.9%)	.035*	.055 (.365—medium)
1	12 (41.4%)	11 (39.3%)	.872	
2	8 (27.6%)	5 (17.9%)	.381	
3	4 (13.8%)	0 (0%)	.042*	
4	0 (0%)	0 (0%)		
	Degenerative patients only (n = 20)	Degenerative patients only (n = 20)		
Stage				
0	5 (25.0%)	9 (45.0%)	.185	.537 (.250—small)
1	10 (50.0%)	8 (40.0%)	.525	
2	4 (20.0%)	3 (15.0%)	.677	
3	1 (5.0%)	0 (0%)	.311	
4	0 (0%)	0 (0%)		
	Deformity patients only (n = 9)	Deformity patients only (n = 8)		
Stage				
0	0 (0%)	3 (37.5%)	.043*	.072 (.634—large)
1	2 (22.2%)	3 (37.5%)	.490	
2	4 (44.4%)	2 (25.0%)	.402	
3	3 (33.3%)	0 (0%)	.072	
4	0 (0%)	0 (0%)		

Data are shown as n (%). *P* values were calculated using Fisher’s exact test. Cramer’s *V* was calculated to determine effect size.

FPU = facial pressure ulcer.

* *P* < .05.

In group A, 4 patients required postoperative plastic surgery consultations. Three patients healed successfully with regular dressing changes and wound care. One patient underwent surgical excision of the necrotic tissue due to a secondary infection at the necrotic site and was subsequently managed for secondary healing. Among the patients in group A who were conservatively monitored following plastic surgery consultation, 1 developed a nasal stricture, necessitating dilation surgery for the nasal passages by the ear, nose, and throat specialist department in the ninth postoperative week. In group B, 1 patient experienced partial laceration of the tongue due to biting and was managed conservatively. Additionally, 1 patient in group A and 4 patients in group B exhibited excessive edema of the lips due to pressure from the endotracheal tube, which resolved with conservative management. Oropharyngeal swelling and macroglossia were observed in 2 patients from each group, all of whom improved with conservative treatment. In group A 1 patient developed total paralysis in the left upper extremity due to excessive extension of the left arm. An MRI performed on the first postoperative day revealed significant brachial plexus edema. By the end of the first year, all findings had resolved, except for partial paralysis of the ulnar nerve. Investigations indicated that the arterial measurement cannula positioned in the left radial artery malfunctioned during surgery, and the anesthesia technician moved the arm into excessive abduction to address the issue, maintaining this position for approximately 4 hours. Notably, no ocular complications were reported in any of the patients.

## 4. Discussion

The primary motivation behind the emergence of this study’s hypothesis was the facial lesions that occur during prolonged prone positioning, and the challenges in managing patients during their recovery. One key insight gained during the study was that the positioning of the patient’s face is as crucial as that of the spine. Appropriate positioning of the thoracic or lumbar spine according to the planned procedure before surgery is essential for spinal surgeons. However, the positioning of the areas outside the patient’s torso often falls outside the surgeon’s focus. Specifically, the head position, typically adjusted by anesthesiologists, can sometimes be overlooked. Although anesthesiologists are knowledgeable about head and face positioning, various factors may distract them during surgery. Therefore, spinal surgeons should pay equal attention to the head position as to the position of the torso, as FPU can lead to significant emotional distress and decreased motivation for recovery, particularly in female patients. In group A, 4 patients required plastic surgery and 1 required intervention by an ear, nose, and throat specialist for postoperative lesions, highlighting prolonged healing times and increased costs as significant issues.

Over time, the elastic properties of prone face pads diminish, and they may be too late to replace them. Numerous types of these pads are available in the market; however, access to quality and new pads is often limited in healthcare facilities. Additionally, surgeons frequently have to use pads available in the hospital, which may not be optimal. Although this study utilized the same pads to enable a standard comparison, we believe that our method is applicable and beneficial for all types of pads. Our initial measure to reduce facial lesions was to wrap the prone pad with 2 layers of cast padding. We hypothesized that friction between the face and pad, as well as the pressure on the face, could be minimized regardless of the pad brand or duration of use. Furthermore, we anticipated that covering the face with a drape would further reduce the adherence and friction at the pad interface, thereby protecting the facial skin. Additionally, changing the location of cream application by the anesthesia team – applying cream over the drape – increases the slipperiness between the drape and pad, thereby reducing friction. Upon implementation, we realized that these 2 methods are practical and time efficient. Evident satisfaction from our initial results was the main driving factor behind this study. If these measures are adopted and learned by anesthesiologists, the reduction of FPU will be facilitated, even if the positioning of the head and the choice of pad escapes the surgeon’s attention.

Two significant findings were obtained from this study. First, in group B, simple measures led to an increase in lesion-free patients, while the number of stage 2 and 3 patients decreased. This finding is particularly important given the prolonged healing times of skin lesions in stage 3 patients and the potential need for plastic surgery consultation. The second important result pertains to the differences between the deformity groups. In patients operated on for ankylosing spondylitis or adult scoliosis, the reduction maneuvers performed during surgery and the elongation of the torso postreduction affect the relationship between the face and pad.^[13]^ Covering the face completely with a drape reduces the adherence and friction between the pad and face during rapid changes related to reduction maneuvers. We also believe that the pressure resulting from the head moving proximally on the pad owing to torso elongation is diminished by the same mechanism. The absence of any stage 3 lesions in the deformity subgroup of group B supports this claim.

A literature review reveals that preventive strategies for complications from the prone facial positioning are highly geared towards preventing ocular complications due to their potential severity.^[3,14–16]^ Cunha et al^[6]^ emphasized the importance for frequent observation of the face and eyes, and recommend that evaluations be made at 15 to 20 minute intervals to determine the lack of compression. While eye health is an ongoing concern, frequent observation facilitates early detection of facial pressure injuries possible as well. Haleem et al^[17]^ identified a number of risk factors for developing iatrogenic facial ulcers and categorizing them as extrinsic and intrinsic factors. Extrinsic factors include prolonged pressure against osseous prominences, shearing and friction, moisture, and material properties existing between skin and headrest. Intrinsic factors comprise advanced age, diabetes mellitus, circulatory compromise, malnutrition, smoking, and medication including corticosteroids. Significantly, prolonged surgical time over 150 minutes increases the risk for developing an ulcer due to repetitive mechanical stress against facial tissues. In light of these risk factors, the authors evaluated an innovative preventive strategy consisting of paraffin tulle gras dressing application over sensitive facial areas (forehead, cheekbones, and chin) on top of a foam facial cushion. The dressing is designed specifically to minimize friction and shearing while maintaining skin integrity.

Prevention of dermal lesions often involves effort at minimizing the time of the surgery and attention to adequate padding and positioning; nevertheless, the broader impact of the surgical modality on patient outcome should be considered.^[1]^

Limitations The current study had several limitations. First, some factors that might have an effect on changing facial pressures during the operation, such as other pathological conditions and body mass index, were not analyzed. However, in order to form a relatively homogeneous study group, the authors excluded patients who were younger, especially with adolescent deformities. Second, the indications and surgical procedures carried out were not analyzed. However, no publication in the literature has ever mentioned that there could be any condition where, due to different indications or surgical technique, the risk for FPU might change. Third, the factors related to anesthesia were not analyzed. However, in order to avoid bias, the same anesthesia protocol was employed in all surgeries, which were carried out by 2 anesthesiologists. Fourth, the small number of patients, especially in the deformity group, is another major limitation. A study conducted with more patients will achieve more reliable results. Lastly, the type of prone pad used can also change the result; therefore, it is also a possibility that group A had better outcomes with a less compressive pad.

In conclusion, wrapping prone pads with cast padding and fully draping the patient’s face is a simple, time-efficient, and universally applicable method that leads to a significant reduction in the FPU associated with prone positioning. Implementation of this as part of routine preparation will reduce the incidence of FPU, enhance patient’s comfort, reduce the costs, and ease the postoperative management.

## Acknowledgments

We would like to thank our nurse Mr Emre Mert for the invaluable contribution he made to coordinate the collection of data on patients and maintain seamless communication between the surgical team and the study participants.

## Author contributions

**Conceptualization:** Mehmet Nuri Erdem.

**Data curation:** Mehmet Nuri Erdem, Yigit Kultur.

**Formal analysis:** Mehmet Nuri Erdem, Yigit Kultur.

**Investigation:** Mehmet Nuri Erdem.

**Methodology:** Mehmet Nuri Erdem, Yigit Kultur.

**Project administration:** Mehmet Nuri Erdem.

**Resources:** Mehmet Nuri Erdem.

**Software:** Mehmet Nuri Erdem, Yigit Kultur.

**Supervision:** Mehmet Nuri Erdem.

**Validation:** Mehmet Nuri Erdem.

**Visualization:** Mehmet Nuri Erdem, Yigit Kultur.

**Writing – original draft:** Mehmet Nuri Erdem, Yigit Kultur.

**Writing – review & editing:** Mehmet Nuri Erdem, Yigit Kultur.
